# Which Method is More Efficient in Determining Osteoporosis, QUS or DEXA?

**DOI:** 10.5812/ijem.5394

**Published:** 2012-09-30

**Authors:** Cem Dane, Banu Dane

**Affiliations:** 1 Department of Gynecology and Obstetrics, Haseki Training and Research Hospital, Istanbul, Turkey; 2 Department of Gynecology and Obstetrics, Bezmialem University, Faculty of Medicine, Istanbul, Turkey

**Keywords:** Osteoporosis, Bone Mineral Density

Dear Editor,

We read with much interest the recently published article, “Effects of Raloxifene on Bone Metabolism in Hemodialysis Patients With Type 2 Diabetes,” by Saito et al (1). First, we would like to congratulate the authors. We wish to share a few scientific facts related to this interesting article.

It was a pleasure to inform from this article that no difference was observed in BMD (bone mineral density) changes between patients with diabetes and nondiabetics after using raloxifene one year long.

We have previously shown that the effects of low dose HRT and raloxifene on BMD at the lumbar spine and hip sites and biochemical markers of bone turnover in a randomized trial (2). In our study, the bone mineral density measurements with dual energy Xray absorptiometry (DEXA) method is accepted by the WHO as the gold standard for the diagnosis of osteoporosis. Satoi et al. proposed the SOS value has been shown to be significantly and positively correlated with lumbar BMD in the general population using methods such as DEXA in Patients and Methods section (1, 3). Quantitative calcaneal ultrasound has several potential advantages, including relatively low cost, no need for ionizing radiation, compact size, and ease of use. These advantages would make Quantitative calcaneal ultrasound an ideal screening tool if it can be shown to be an accurate indicator of skeletal density and fracture risk (4). The BMD measurement is accepted by the WHO as the gold standard for the diagnosis of osteoporosis. In another study, we have found a poor to moderate correlation between Quantitative ultrasound parameters (BUA, SOS, SI) and femoral and spinal BMD in pre and postmenopausal women (5). Tuna et al also reported, that the diagnostic value of tibial SOS did not seem to be high for discriminating between normal and low BMD at the femur and lumbar spine in postmenopausal women (6).

I think that their results would be more precise if the authors’ preferred DEXA in spite of the measurement of calcaneal quantitative ultrasound parameters (SOS).

We appreciate the authors and editor for publishing an important clinical article that may be beneficial to clinicians.
